# Static and dynamic 3D culture of neural precursor cells on macroporous cryogel microcarriers

**DOI:** 10.1016/j.mex.2020.100805

**Published:** 2020-01-23

**Authors:** Ben Newland, Fanny Ehret, Franziska Hoppe, Dimitri Eigel, Dagmar Pette, Heike Newland, Petra B. Welzel, Gerd Kempermann, Carsten Werner

**Affiliations:** a Leibniz Institute of Polymer Research Dresden, Max Bergmann Center of Biomaterials Dresden, 01069, Dresden, Germany; b School of Pharmacy and Pharmaceutical Sciences, Cardiff University, CF10 3NB, Cardiff, UK; c German Center for Neurodegenerative Diseases (DZNE) Dresden, 01307, Dresden, Germany; d CRTD – Center for Regenerative Therapies Dresden, Technische Universität Dresden, 01307, Dresden, Germany

**Keywords:** 3D culture of neural precursor cells on macroporous cryogel microcarriers, Scaffolds, Neural progenitor differentiation, Cell survival, Dentate gyrus, Heparin, Biomaterials for cell culture

## Abstract

Neural precursor cells have been much studied to further our understanding of the far-reaching and controversial question of adult neurogenesis. Currently, differentiation of primary neural precursor cells from the mouse dentate gyrus via 2-dimentional *in vitro* culture yields low numbers of neurons, a major hindrance to the field of study. 3-dimentional “neurosphere” culture allows better 3D cell-cell contact, but control over cell differentiation is poor because nutrition and oxygen restrictions at the core of the sphere causes spontaneous differentiation, predominantly to glial cells, not neurons. Our group has developed macroporous scaffolds, which overcome the above-mentioned problems, allowing long-term culture of neural stem cells, which can be differentiated into a much higher yield of neurons. Herein we describe a method for culturing neural precursor cells on RGD peptide functionalized-heparin containing cryogel scaffolds, either in standard non-adherent well-plates (static culture) or in spinner flasks (dynamic culture). This method includes:

• The synthesis and characterization of heparin based microcarriers.
• A “static” 3D culture method for that does not require spinner flask equipment.
• “Dynamic” culture in which cell loaded microcarriers are transferred to a spinner flask.

## Graphical abstract

**Figure:**
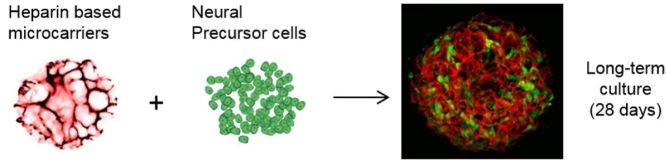

## Specification Table

**Table:** 

Subject Area:	Neuroscience
More specific subject area:	*Biomaterials for neural cell culture*
Method name:	*3D culture of neural precursor cells on macroporous cryogel microcarriers*
Name and reference of original method:	*B. Newland, F. Ehret, F. Hoppe, D. Eigel, D. Pette, H. Newland, P. B. Welzel, G. Kempermann, C. Werner, Macroporous heparin-based microcarriers allow long-term 3D culture and differentiation of neural precursor cells, Biomaterials, 2019* *B. Newland, P. B. Welzel, H. Newland, C. Renneberg, P. Kolar, M. Tsurkan, A. Rosser, U. Freudenberg, C. Werner, Tackling Cell Transplantation Anoikis: An Injectable, Shape Memory Cryogel Microcarrier Platform Material for Stem Cell and Neuronal Cell Growth, Small, 2015, 11, 5047-5053*
Resource availability:	Raw data are available upon request.

## Method details

### Cryogel microcarrier preparation and biofunctionalization

The cryogel microcarriers were prepared in a manner similar to that reported previously [1]. For preparation of the hydrogel precursor solution a molar ratio of four-arm amino-terminated PEG (10 kDa, JenKem Technology USA) to heparin (14 kDa, Sodium Salt, Porcine Intestinal Mucosa, Calbiochem, Merck) of 1:1 was used. A molar ratio of 1-ethyl-3-(3-dimethylaminopropyl)carbodiimide (EDC, Sigma-Aldrich) to N-hydroxysulfosuccinimide sodium salt (sulfo-NHS, Sigma) of 2:1 was used. 1 % of the heparin used in the cryogel microcarrier preparation was Alexa 647 labelled (prepared from Alexa 647, Invitrogen as reported previously [1]). All components were dissolved in deionized, decarbonized water (MilliQ) by vortexing and ultrasonication and were kept on ice afterwards. Activation of carboxylic acid groups of heparin was performed by mixing the EDC, sulfo-NHS and heparin solutions and leaving them on ice for 15 min. Then the PEG solution was added and 600 μL of this final precursor solution were immediately injected into a mixture of 9 mL toluene and 3 mL of a Synperonic PEP105 (Sigma-Aldrich) in toluene solution (c =1 mg/mL, as a stabilizing agent). The resulting emulsion was stirred at 700 rpm for 10 min at room temperature for droplet formation to occur, before another 3 mL of Synperonic PEP105 in toluene solution (1 mg/mL) were added to the stirring solution to avoid droplet agglomeration. Subsequently, the flask containing the emulsion was immersed into a −80 °C ethanol bath for 2 h. The resulting microcarriers were kept at −20 °C over-night and subsequently lyophilized for at least 24 h, before they were dispersed in ethanol and afterwards washed several times with MilliQ water.

The microcarriers were used as such (without RGD) or they were functionalized with a cyclic peptide sequence containing the RGD motif (cyclo(Arg-Gly-Asp-D-Tyr-Lys) PCI-3662-PI, (Peptides International). For RDG-functionalization 5 mg of dry microcarriers were activated by sterile filtered EDC (28.8 mg) and sulfo-NHS (16.3 mg) (molar ratio of 2:1) solution in 1.5 mL phosphate buffer (pH = 5) for 45 min at 4 °C while shaking at 400 rpm. The buffer was exchanged to borate buffer (pH = 8) before adding a 200 μg/mL solution of the cyclic peptide dissolved in borate buffer. Incubation lasted for 2 h at room temperature shaking at 400 rpm. Functionalized microcarriers were washed with MilliQ water three times, frozen in liquid nitrogen and lyophilized.

### Microcarrier characterization

The dried microcarriers were attached to the sample holder via carbon adhesive, and sputtered with gold for 60 s at 40 mA (SCD 050 Sputter Coater, Balzers). These samples were then imaged using a XL30 ESEM-FEG scanning electron microscope (Philips) using the secondary electron detector and acceleration voltages of 3.0–5.0 kV. A Dragonfly spinning disc confocal laser microscope (SPCLM) (Andor Technology Ltd, Belfast, Ireland) mounted on a Nikon Ti-E inverted microscope was used to visualize the Atto 647 labelled microcarriers in the hydrated state (phosphate-buffered saline solution (PBS)), with a 637 nm laser diode and images were taken with a 10x magnification objective (CFI Plan Apo Lambda, Nikon). Microcarrier diameter and pore size were determined using ImageJ software (NIH) as described previously [1,2].

### Neural precursor cell (NPC) culture

Hippocampal neural precursor cells (NPCs) extracted from the dentate gyri of 6–8 week old C57BL/6 mice (as previously reported [3]). For culture in non-differentiating (proliferative) conditions, NPC were cultivated for up to 7 passages on poly-D-lysine and laminin coated flasks in neurobasal medium (NB medium, Gibco) supplemented with B27 (Gibco), glutamax (ThermoFisher) (2 mM end concentration), human epidermal growth factor (EGF, Peprotech) (10 ng/mL) and fibroblast growth factor (bFGF, Peprotech) (10 ng/mL) (NB + B27 medium) at 37 °C in 5 % CO_2_ at 90 % humidity [4]. The medium was changed every other day.

#### D NPC culture

For 2D NPC experiments 40,000 cells were seeded on laminin and poly-D-lysine coated 12 mm glass cover slips and allowed to proliferate until 80 % confluency was reached. Part of this culture was fixed with 4 % paraformaldehyde (PFA) in PBS for 10 min and stored in Dulbecco´s phosphate-buffered saline already containing calcium and magnesium (DPBS, ThermoFisher Ref. 4080055) at 4 °C until stained. In parallel, the non-fixed cells in 2D culture were differentiated via the withdrawal of growth factors. NPCs were differentiated for 3 and 6 days and subsequently fixed with 4 % PFA in PBS for 10 min and stored in DPBS at 4 °C until staining was carried out.

#### D NPC culture on the microcarriers

**Fig. 1:**
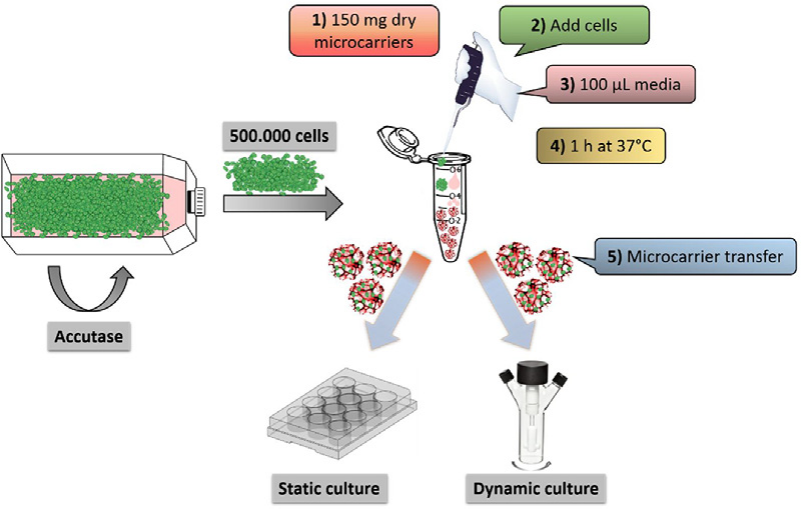
A schematic representation of the 3D culture protocol whereby the dry microcarriers are first loaded in a centrifuge tube before transfer to the desired culture conditions.

NPCs were removed from the 2D culture flask by incubation with Accutase (Sigma-Aldrich) for 3 min at 37 °C. 500,000 cells were pipetted onto 1.5 mg of dry RGD-functionalized microcarriers in a centrifuge tube and topped up with 100 μL of NB + B27 medium. The cell loaded microcarriers were incubated for 1 h at 37 °C before being transferred to a 12-well plate for non-adherent culture (static culture) or a Sigmacote (Sigma-Aldrich) treated spinner flask (dynamic culture) (Fig. 1). Wells contained 1 mL of medium or spinner flasks (GPE Scientific Ltd – standard 25 mL flat bottom flask) contained 25 mL of medium (medium = NB + B27 media with growth factors EGF and bFGF as described above). A spin speed of 80 rpm was used, controlled by a Cimarec i Poly 15 stir plate (ThermoFisher). For proliferation conditions, half of the medium was changed every other day. Analysis was performed after 3, 7, 14 and 28 days. Differentiation of the 3D culture was induced by withdrawal of growth factors and samples were analyzed after a further 1, 3, 6 and 14 days. For 3D culture experiments on Cultispher S (Sigma-Aldrich), the Cultisphers were first sterilized and dried according to the manufacturer’s protocol, and used in an identical manner to the cryogel microcarriers.

### Immunocytochemistry staining

2D NPC cultures and cells on microcarriers were fixed with 4 % PFA in PBS, permeabilized with 0.1 % Triton-X100 (Sigma) for 15 min, blocked with 10 % donkey serum (Dianova, Ref. 017-000-121) for 30 min. Subsequently, cells were incubated with either rabbit anti-Sox2 (Millipore, AB5603, 1:400) and mouse anti-nestin (BD Biosciences, 611658, 1:250), rabbit anti-PCNA (abcam, ab18197, 1:1000) and mouse anti-nestin (BD Biosciences, 611658, 1:250) or rabbit anti-GFAP (Dako, Z0334, 1:250) and mouse anti-Map2 (Sigma, M1406 in. 1:1000) in blocking solution at 4 °C over-night. After washing with 1X DPBS three times, secondary antibody incubation was performed with donkey anti-rabbit Alexa Fluor568 IgG (H + L) (Life technologies, A10042, 1:500) and donkey anti-mouse Alexa Fluor488 IgG (H + L) (Dianova, 715-545-151, 1:500) in blocking solution for 3 h at room temperature in the dark. Unbound secondary antibody was washed away with 1X DPBS. Thereafter, cells were counterstained with Hoechst 33342 nuclear dye (Molecular Probes, Invitrogen, 1:1000 in DPBS) for 10 min and again washed with 1X DPBS.

### Live-dead staining

To determine the percentage of dead cells, a live-dead solution calcein AM Solution (PromoKine) and propidium iodide (Sigma) was added to cells cultured on microcarriers in NB + B27 medium and incubated for 20 min at 37 °C. Cells were counterstained with Hoechst 33342 1:1000 in DPBS for 10 min and washed with NB medium once. For the cell harvesting experiment, microcarriers with cells were pelleted at 50 g for 1 min, followed by incubation in 500 μl accumax or accutase for 5 min. The cells were washed once in PBS, and then resuspended in PBS containing trypan blue and analyzed using a Countess 2 cell counter (Life Technologies). The number and percentage of viable cells was counted. The microcarriers were then analyzed separately by qualitative confocal microscopy.

### Microscopy analysis

Immediately after live-dead staining cells were imaged with a Dragonfly spinning disc confocal laser microscope at 20x magnification. Per culture condition 15 z-stacks of 40 μm depth with a 1 μm step size were taken for quantitative analysis. For qualitative images, 40 μm and 80 μm z-stacks with step sizes of 0.5 μm were acquired. For each microcarrier a single image of the cross section was taken to determine its diameter. Analysis of pictures was performed using ImageJ (NIH). For quantitative analysis images were processed by subtracting the background with a rolling ball radius of 5 pixels. Cell nuclei were counted automatically by 3D objects counter v2.0 with a size filter set to 100 minimum. Mean values for the total amount of cells, the amount of dead cells, the number of Sox2 and PCNA positive cells were determined. Map2 and GFAP positive cells were counted manually. Ratios of amount of dead cells, Sox2, PCNA, MAP2 or GFAP positive cells to total amount of cells per microcarrier were calculated and plotted. For qualitative analysis, the microscopy images were processed with Imaris software (Bitplane).

### Statistical analysis

Microsoft Excel was used to determine the R2 values and Graph Pad Prism software was used to perform the unpaired *t*-test analysis comparing differentiation conditions to the equivalent proliferation conditions. The same test was used for the comparison between microcarrier and Cultispher culture. Three replicates were carried out for each study and the total number of microcarriers and the total number of cells analyzed for each condition is indicated in the figure legends in the corresponding Biomaterials publication [5].

### Method validation

**Fig. 2:**
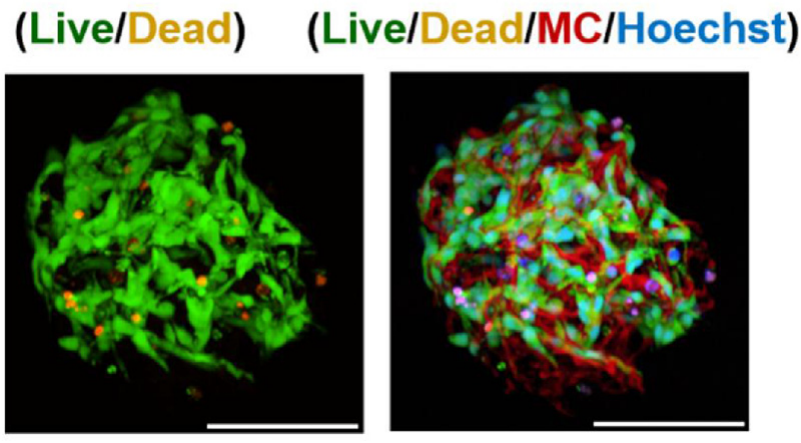
A representative fluorescent microscope image (40 μm Z-projection) of neural precursor cells analyzed by the Live/Dead cell assay after three days of static culture on a microcarrier. Live cells (green) can be seen adhered to the microcarrier (red) with nuclear counterstaining (blue) and few dead cells (orange). Such analysis can be used to validate the growth of the cells within the pores of the microcarriers. Scale bars represent 100 μm. See the parallel publication for further microscopy analysis [5].

Microcarriers were characterized in terms of size and pore size and live dead analysis of neural precursor cells cultured on the microcarriers was carried out in proliferative and differentiation conditions [5]. Fig. 2 shows the result of live/dead cell analysis of neural precursor cells cultured on a microcarrier for three days in proliferative conditions, showing cells attached to the struts of the microcarrier structure with few dead cells.
